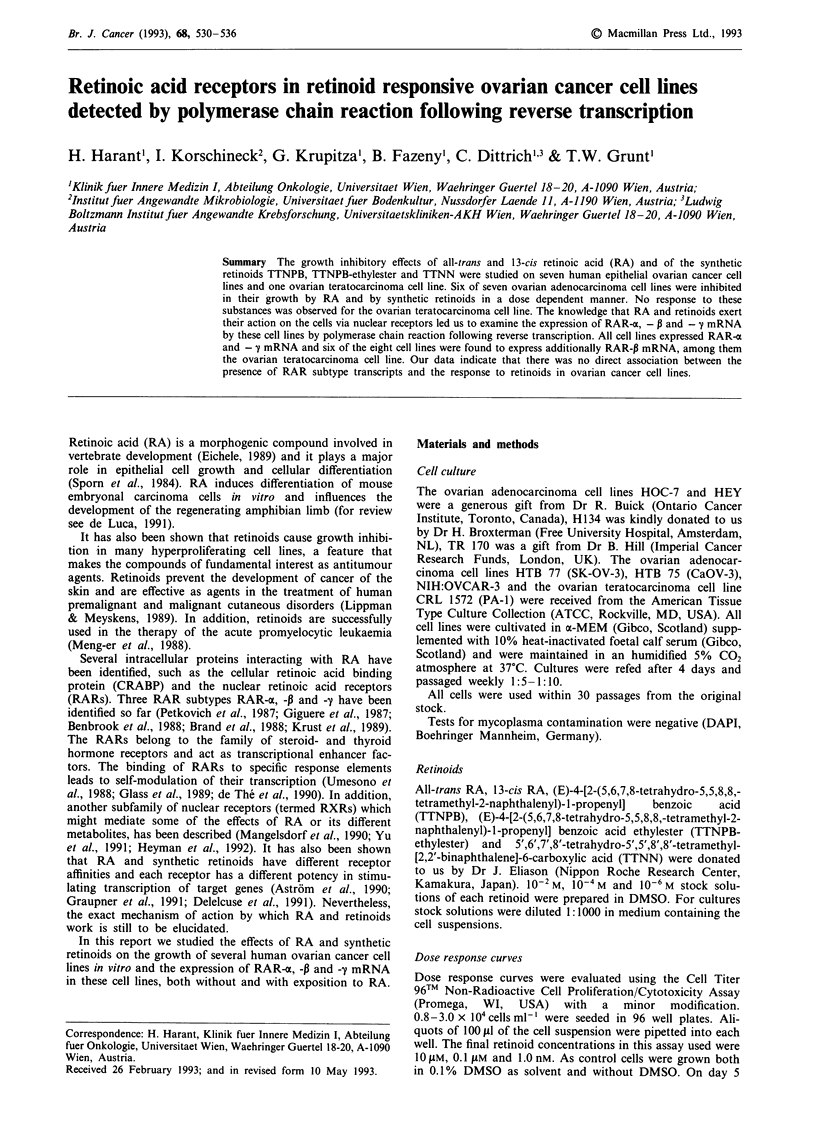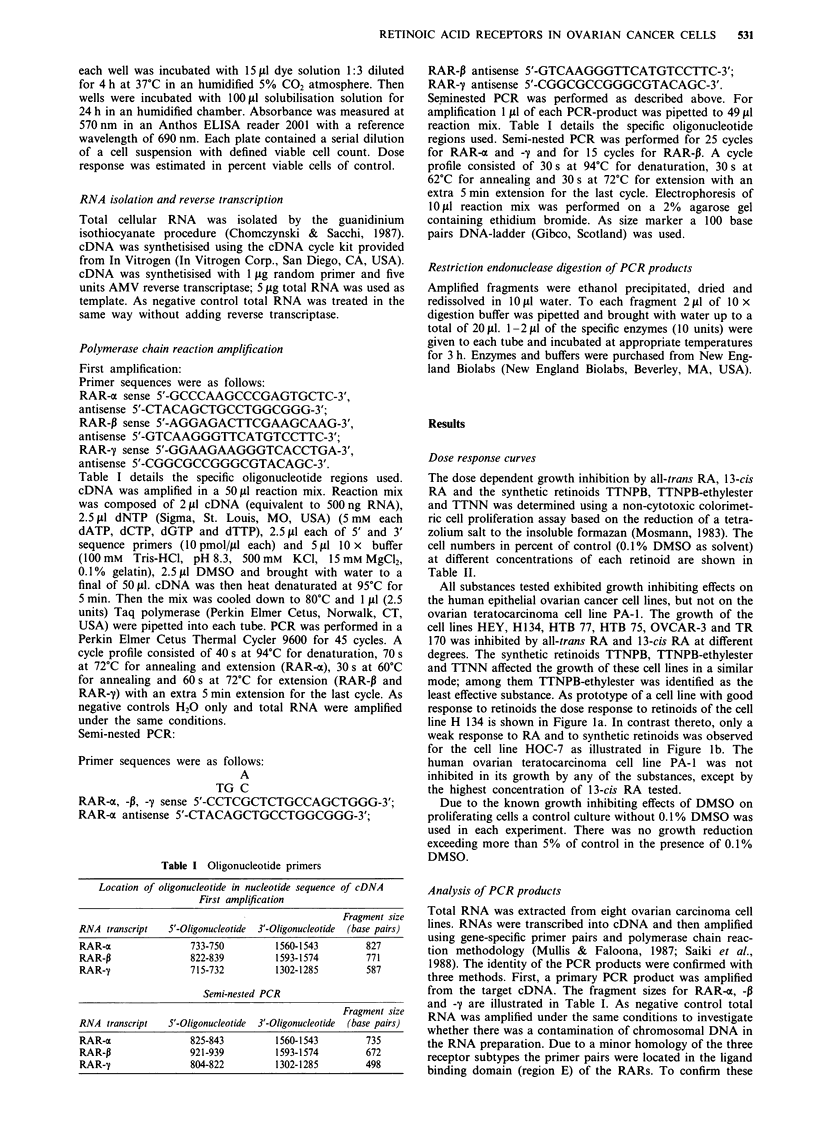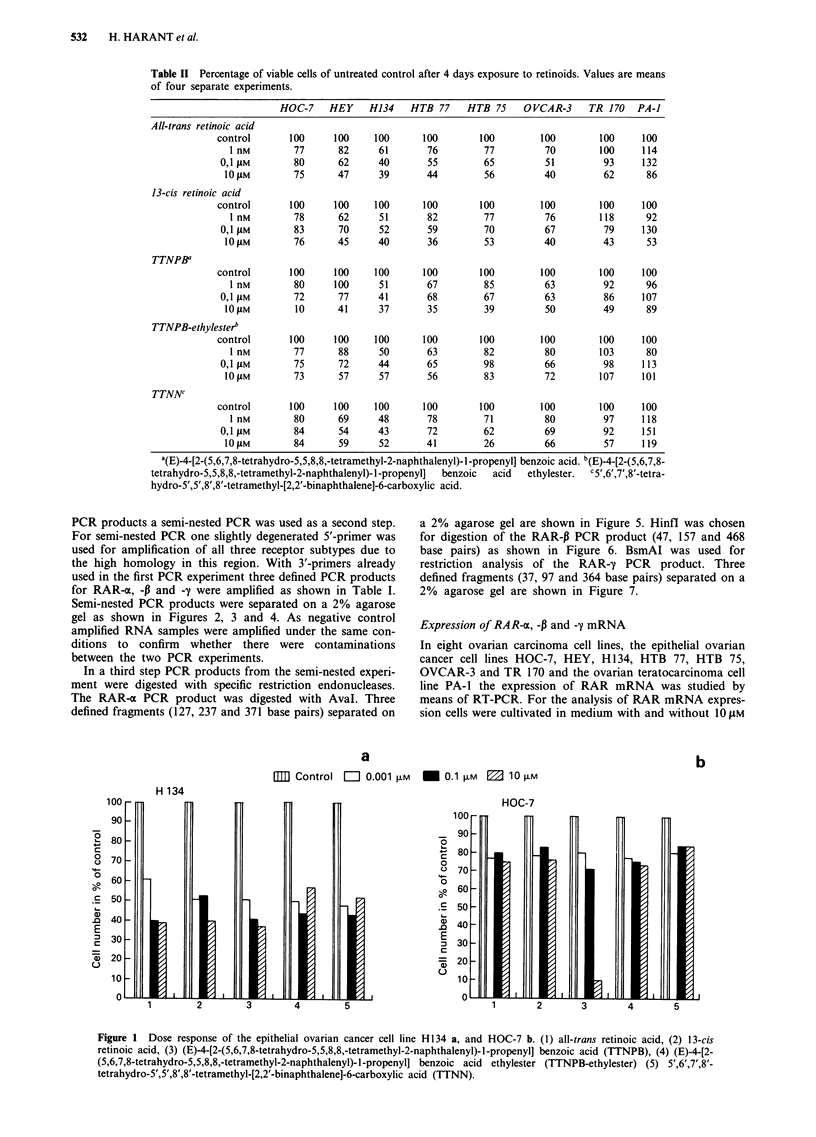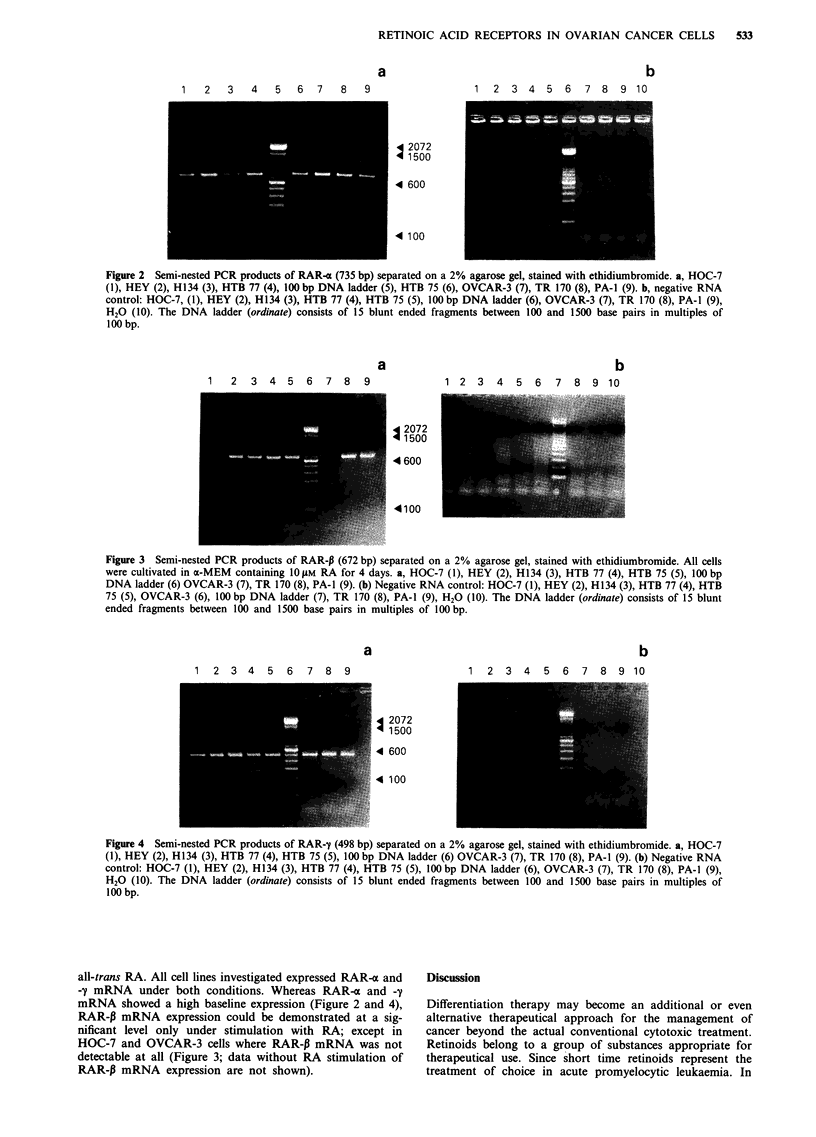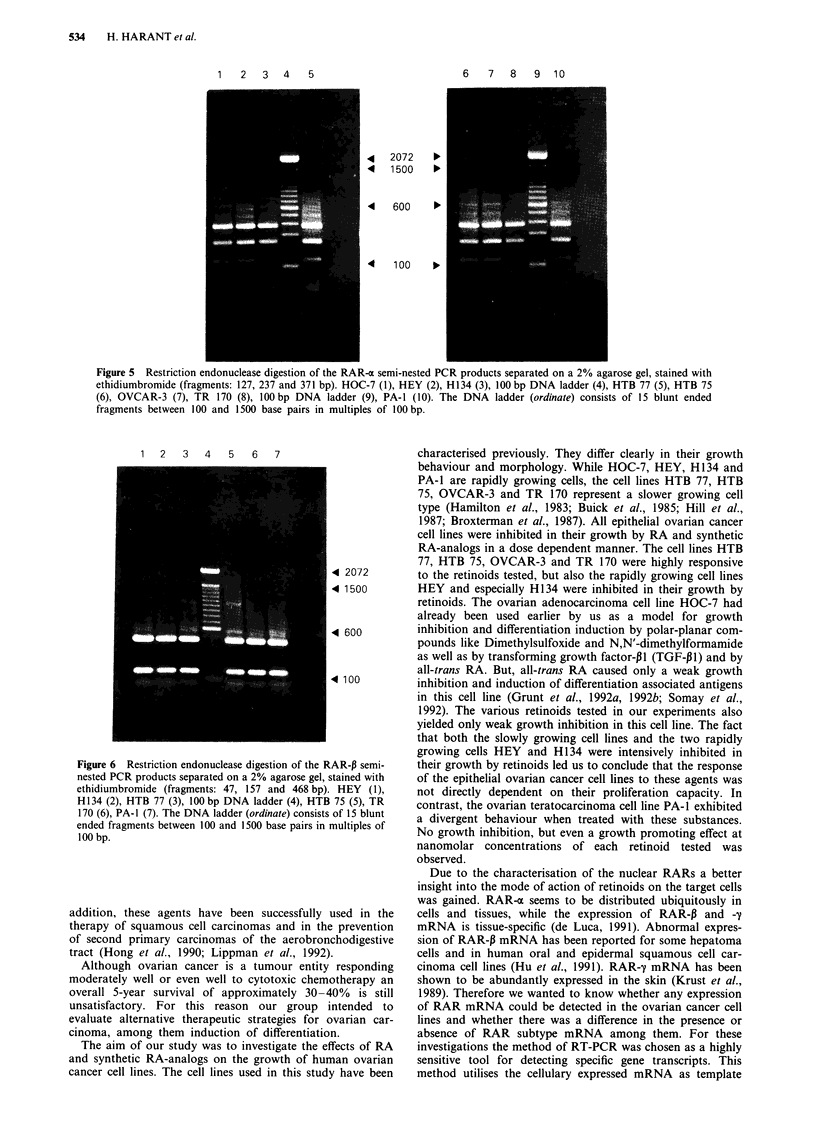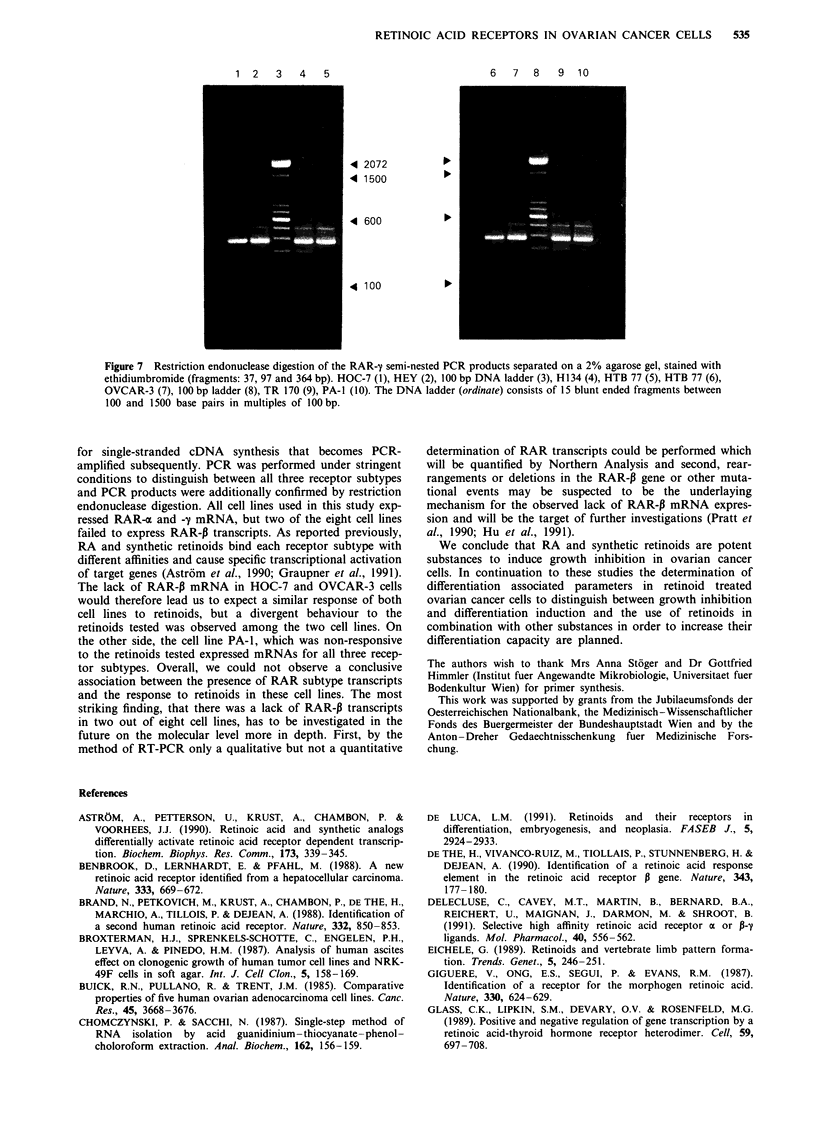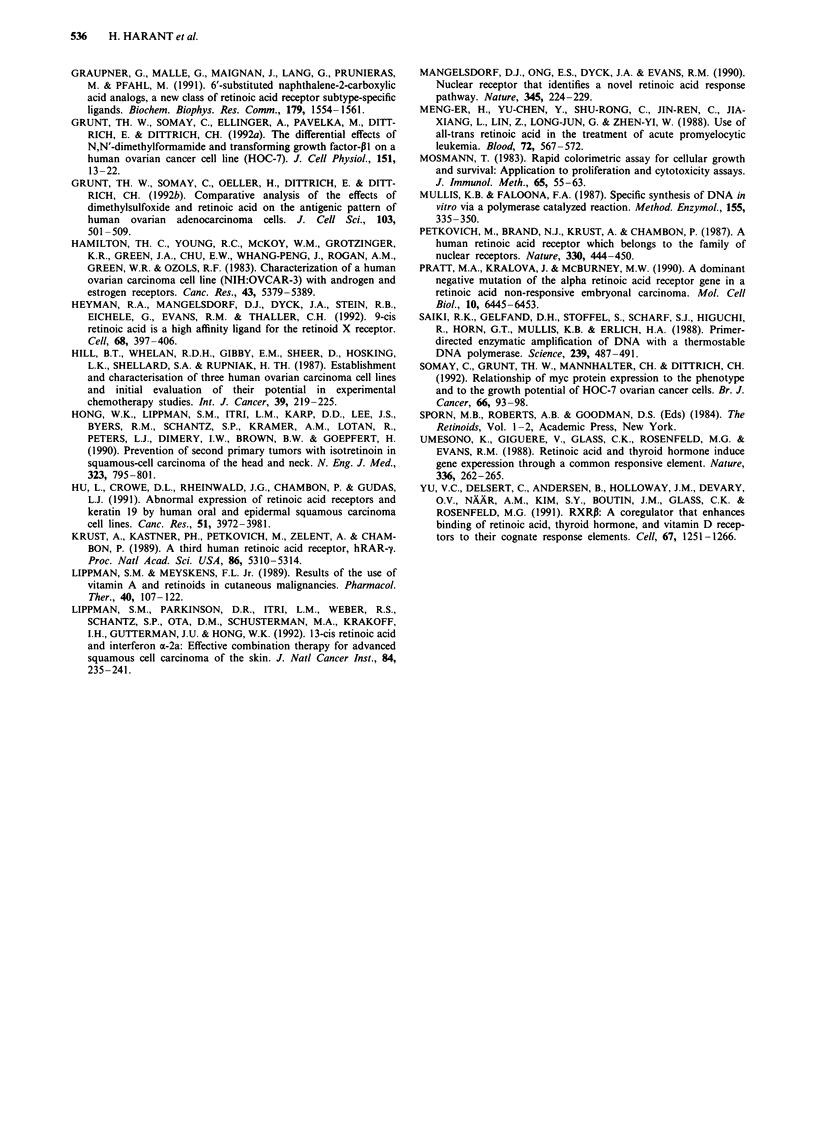# Retinoic acid receptors in retinoid responsive ovarian cancer cell lines detected by polymerase chain reaction following reverse transcription.

**DOI:** 10.1038/bjc.1993.381

**Published:** 1993-09

**Authors:** H. Harant, I. Korschineck, G. Krupitza, B. Fazeny, C. Dittrich, T. W. Grunt

**Affiliations:** Abteilung Onkologie, Universitaet Wien, Austria.

## Abstract

**Images:**


Br. J. Cancer (1993), 68, 530-536 © Macmillan Press Ltd., 1993

### Retinoic acid receptors in retinoid responsive ovarian cancer cell lines detected by polymerase chain reaction following reverse transcription

H. Harant[1], I. Korschineck[2], G. Krupitza[1], B. Fazeny[1], C. Dittrich[1,3] & T.W. Grunt[1]

[1]*Klinik fuer Innere Medizin I, Abteilung Onkologie, Universitaet Wien, Waehringer Guertel 18-20, A-1090 Wien, Austria;* [2]*Institut fuer Angewandte Mikrobiologie, Universitaet fuer Bodenkultur, Nussdorfer Laende 11, A-1190 Wien, Austria;* [3]*Ludwig Boltzmann Institut fuer Angewandte Krebsforschung, Universitaetskliniken-AKH Wien, Waehringer Guertel 18-20, A-1090 Wien, Austria*

**Summary** The growth inhibitory effects of all-*trans* and 13-*cis* retinoic acid (RA) and of the synthetic retinoids TTNPB, TTNPB-ethylester and TTNN were studied on seven human epithelial ovarian cancer cell lines and one ovarian teratocarcinoma cell line. Six of seven ovarian adenocarcinoma cell lines were inhibited in their growth by RA and by synthetic retinoids in a dose dependent manner. No response to these substances was observed for the ovarian teratocarcinoma cell line. The knowledge that RA and retinoids exert their action on the cells via nuclear receptors led us to examine the expression of RAR-α, − β and − γ mRNA by these cell lines by polymerase chain reaction following reverse transcription. All cell lines expressed RAR-α and − γ mRNA and six of the eight cell lines were found to express additionally RAR-β mRNA, among them the ovarian teratocarcinoma cell line. Our data indicate that there was no direct association between the presence of RAR subtype transcripts and the response to retinoids in ovarian cancer cell lines.

Retinoic acid (RA) is a morphogenic compound involved in vertebrate development (Eichele, 1989) and it plays a major role in epithelial cell growth and cellular differentiation (Sporn *et al.*, 1984). RA induces differentiation of mouse embryonal carcinoma cells *in vitro* and influences the development of the regenerating amphibian limb (for review see de Luca, 1991).

It has also been shown that retinoids cause growth inhibition in many hyperproliferating cell lines, a feature that makes the compounds of fundamental interest as antitumour agents. Retinoids prevent the development of cancer of the skin and are effective as agents in the treatment of human premalignant and malignant cutaneous disorders (Lippman & Meyskens, 1989). In addition, retinoids are successfully used in the therapy of the acute promyelocytic leukaemia (Meng-er *et al.*, 1988).

Several intracellular proteins interacting with RA have been identified, such as the cellular retinoic acid binding protein (CRABP) and the nuclear retinoic acid receptors (RARs). Three RAR subtypes RAR-α, -β and -γ have been identified so far (Petkovich *et al.*, 1987; Giguere *et al.*, 1987; Benbrook *et al.*, 1988; Brand *et al.*, 1988; Krust *et al.*, 1989). The RARs belong to the family of steroid- and thyroid hormone receptors and act as transcriptional enhancer factors. The binding of RARs to specific response elements leads to self-modulation of their transcription (Umesono *et al.*, 1988; Glass *et al.*, 1989; de Thé *et al.*, 1990). In addition, another subfamily of nuclear receptors (termed RXRs) which might mediate some of the effects of RA or its different metabolites, has been described (Mangelsdorf *et al.*, 1990; Yu *et al.*, 1991; Heyman *et al.*, 1992). It has also been shown that RA and synthetic retinoids have different receptor affinities and each receptor has a different potency in stimulating transcription of target genes (Aström *et al.*, 1990; Graupner *et al.*, 1991; Delelcuse *et al.*, 1991). Nevertheless, the exact mechanism of action by which RA and retinoids work is still to be elucidated.

In this report we studied the effects of RA and synthetic retinoids on the growth of several human ovarian cancer cell lines *in vitro* and the expression of RAR-α, -β and -γ mRNA in these cell lines, both without and with exposition to RA.

Correspondence: H. Harant, Klinik fuer Innere Medizin I, Abteilung fuer Onkologie, Universitaet Wien, Waehringer Guertel 18-20, A-1090 Wien, Austria.
Received 26 February 1993; and in revised form 10 May 1993.

### Materials and methods

### *Cell culture*

The ovarian adenocarcinoma cell lines HOC-7 and HEY were a generous gift from Dr R. Buick (Ontario Cancer Institute, Toronto, Canada), H134 was kindly donated to us by Dr H. Broxterman (Free University Hospital, Amsterdam, NL), TR 170 was a gift from Dr B. Hill (Imperial Cancer Research Funds, London, UK). The ovarian adenocarcinoma cell lines HTB 77 (SK-OV-3), HTB 75 (CaOV-3), NIH:OVCAR-3 and the ovarian teratocarcinoma cell line CRL 1572 (PA-1) were received from the American Tissue Type Culture Collection (ATCC, Rockville, MD, USA). All cell lines were cultivated in α-MEM (Gibco, Scotland) supplemented with 10% heat-inactivated foetal calf serum (Gibco, Scotland) and were maintained in an humidified 5% $CO_2$ atmosphere at 37°C. Cultures were refed after 4 days and passaged weekly 1:5-1:10.

All cells were used within 30 passages from the original stock.

Tests for mycoplasma contamination were negative (DAPI, Boehringer Mannheim, Germany).

### *Retinoids*

All-*trans* RA, 13-*cis* RA, (E)-4-[2-(5,6,7,8-tetrahydro-5,5,8,8,-tetramethyl-2-naphthalenyl)-1-propenyl] benzoic acid (TTNPB), (E)-4-[2-(5,6,7,8-tetrahydro-5,5,8,8,-tetramethyl-2-naphthalenyl)-1-propenyl] benzoic acid ethylester (TTNPB-ethylester) and 5′,6′,7′,8′-tetrahydro-5′,5′,8′,8′-tetramethyl-[2,2′-binaphthalene]-6-carboxylic acid (TTNN) were donated to us by Dr J. Eliason (Nippon Roche Research Center, Kamakura, Japan). $10^{-2}$ M, $10^{-4}$ M and $10^{-6}$ M stock solutions of each retinoid were prepared in DMSO. For cultures stock solutions were diluted 1:1000 in medium containing the cell suspensions.

### *Dose response curves*

Dose response curves were evaluated using the Cell Titer 96™ Non-Radioactive Cell Proliferation/Cytotoxicity Assay (Promega, WI, USA) with a minor modification. $0.8-3.0 \times 10^4$ cells $ml^{-1}$ were seeded in 96 well plates. Aliquots of 100 μl of the cell suspension were pipetted into each well. The final retinoid concentrations in this assay used were 10 μM, 0.1 μM and 1.0 nM. As control cells were grown both in 0.1% DMSO as solvent and without DMSO. On day 5

each well was incubated with 15 μl dye solution 1:3 diluted for 4 h at 37°C in an humidified 5% $CO_2$ atmosphere. Then wells were incubated with 100 μl solubilisation solution for 24 h in an humidified chamber. Absorbance was measured at 570 nm in an Anthos ELISA reader 2001 with a reference wavelength of 690 nm. Each plate contained a serial dilution of a cell suspension with defined viable cell count. Dose response was estimated in percent viable cells of control.

*RNA isolation and reverse transcription*

Total cellular RNA was isolated by the guanidinium isothiocyanate procedure (Chomczynski & Sacchi, 1987). cDNA was synthetisised using the cDNA cycle kit provided from In Vitrogen (In Vitrogen Corp., San Diego, CA, USA). cDNA was synthetisised with 1 μg random primer and five units AMV reverse transcriptase; 5 μg total RNA was used as template. As negative control total RNA was treated in the same way without adding reverse transcriptase.

*Polymerase chain reaction amplification*

First amplification:
Primer sequences were as follows:
RAR-α sense 5′-GCCCAAGCCCGAGTGCTC-3′,
antisense 5′-CTACAGCTGCCTGGCGGG-3′;
RAR-β sense 5′-AGGAGACTTCGAAGCAAG-3′,
antisense 5′-GTCAAGGGTTCATGTCCTTC-3′;
RAR-γ sense 5′-GGAAGAAGGGTCACCTGA-3′,
antisense 5′-CGGCGCCGGGCGTACAGC-3′.
Table I details the specific oligonucleotide regions used. cDNA was amplified in a 50 μl reaction mix. Reaction mix was composed of 2 μl cDNA (equivalent to 500 ng RNA), 2.5 μl dNTP (Sigma, St. Louis, MO, USA) (5 mM each dATP, dCTP, dGTP and dTTP), 2.5 μl each of 5′ and 3′ sequence primers (10 pmol/μl each) and 5 μl 10 × buffer (100 mM Tris-HCl, pH 8.3, 500 mM KCl, 15 mM $MgCl_2$, 0.1% gelatin), 2.5 μl DMSO and brought with water to a final of 50 μl. cDNA was then heat denaturated at 95°C for 5 min. Then the mix was cooled down to 80°C and 1 μl (2.5 units) Taq polymerase (Perkin Elmer Cetus, Norwalk, CT, USA) were pipetted into each tube. PCR was performed in a Perkin Elmer Cetus Thermal Cycler 9600 for 45 cycles. A cycle profile consisted of 40 s at 94°C for denaturation, 70 s at 72°C for annealing and extension (RAR-α), 30 s at 60°C for annealing and 60 s at 72°C for extension (RAR-β and RAR-γ) with an extra 5 min extension for the last cycle. As negative controls $H_2O$ only and total RNA were amplified under the same conditions.
Semi-nested PCR:

Primer sequences were as follows:
A
TG C
RAR-α, -β, -γ sense 5′-CCTCGCTCTGCCAGCTGGG-3′;
RAR-α antisense 5′-CTACAGCTGCCTGGCGGG-3′;
RAR-β antisense 5′-GTCAAGGGTTCATGTCCTTC-3′;
RAR-γ antisense 5′-CGGCGCCGGGCGTACAGC-3′.
Seminested PCR was performed as described above. For amplification 1 μl of each PCR-product was pipetted to 49 μl reaction mix. Table I details the specific oligonucleotide regions used. Semi-nested PCR was performed for 25 cycles for RAR-α and -γ and for 15 cycles for RAR-β. A cycle profile consisted of 30 s at 94°C for denaturation, 30 s at 62°C for annealing and 30 s at 72°C for extension with an extra 5 min extension for the last cycle. Electrophoresis of 10 μl reaction mix was performed on a 2% agarose gel containing ethidium bromide. As size marker a 100 base pairs DNA-ladder (Gibco, Scotland) was used.

*Restriction endonuclease digestion of PCR products*

Amplified fragments were ethanol precipitated, dried and redissolved in 10 μl water. To each fragment 2 μl of 10 × digestion buffer was pipetted and brought with water up to a total of 20 μl. 1–2 μl of the specific enzymes (10 units) were given to each tube and incubated at appropriate temperatures for 3 h. Enzymes and buffers were purchased from New England Biolabs (New England Biolabs, Beverley, MA, USA).

**Table I** Oligonucleotide primers

| *Location of oligonucleotide in nucleotide sequence of cDNA* | | | |
|---|---|---|---|
| *First amplification* | | | |
| *RNA transcript* | *5′-Oligonucleotide* | *3′-Oligonucleotide* | *Fragment size (base pairs)* |
| RAR-α | 733-750 | 1560-1543 | 827 |
| RAR-β | 822-839 | 1593-1574 | 771 |
| RAR-γ | 715-732 | 1302-1285 | 587 |
| *Semi-nested PCR* | | | |
| *RNA transcript* | *5′-Oligonucleotide* | *3′-Oligonucleotide* | *Fragment size (base pairs)* |
| RAR-α | 825-843 | 1560-1543 | 735 |
| RAR-β | 921-939 | 1593-1574 | 672 |
| RAR-γ | 804-822 | 1302-1285 | 498 |

### Results

*Dose response curves*

The dose dependent growth inhibition by all-*trans* RA, 13-*cis* RA and the synthetic retinoids TTNPB, TTNPB-ethylester and TTNN was determined using a non-cytotoxic colorimetric cell proliferation assay based on the reduction of a tetrazolium salt to the insoluble formazan (Mosmann, 1983). The cell numbers in percent of control (0.1% DMSO as solvent) at different concentrations of each retinoid are shown in Table II.

All substances tested exhibited growth inhibiting effects on the human epithelial ovarian cancer cell lines, but not on the ovarian teratocarcinoma cell line PA-1. The growth of the cell lines HEY, H134, HTB 77, HTB 75, OVCAR-3 and TR 170 was inhibited by all-*trans* RA and 13-*cis* RA at different degrees. The synthetic retinoids TTNPB, TTNPB-ethylester and TTNN affected the growth of these cell lines in a similar mode; among them TTNPB-ethylester was identified as the least effective substance. As prototype of a cell line with good response to retinoids the dose response to retinoids of the cell line H 134 is shown in Figure 1a. In contrast thereto, only a weak response to RA and to synthetic retinoids was observed for the cell line HOC-7 as illustrated in Figure 1b. The human ovarian teratocarcinoma cell line PA-1 was not inhibited in its growth by any of the substances, except by the highest concentration of 13-*cis* RA tested.

Due to the known growth inhibiting effects of DMSO on proliferating cells a control culture without 0.1% DMSO was used in each experiment. There was no growth reduction exceeding more than 5% of control in the presence of 0.1% DMSO.

*Analysis of PCR products*

Total RNA was extracted from eight ovarian carcinoma cell lines. RNAs were transcribed into cDNA and then amplified using gene-specific primer pairs and polymerase chain reaction methodology (Mullis & Faloona, 1987; Saiki *et al.*, 1988). The identity of the PCR products were confirmed with three methods. First, a primary PCR product was amplified from the target cDNA. The fragment sizes for RAR-α, -β and -γ are illustrated in Table I. As negative control total RNA was amplified under the same conditions to investigate whether there was a contamination of chromosomal DNA in the RNA preparation. Due to a minor homology of the three receptor subtypes the primer pairs were located in the ligand binding domain (region E) of the RARs. To confirm these

**Table II** Percentage of viable cells of untreated control after 4 days exposure to retinoids. Values are means of four separate experiments.

| | HOC-7 | HEY | H134 | HTB 77 | HTB 75 | OVCAR-3 | TR 170 | PA-1 |
|---|---|---|---|---|---|---|---|---|
| *All-trans retinoic acid* | | | | | | | | |
| control | 100 | 100 | 100 | 100 | 100 | 100 | 100 | 100 |
| 1 nM | 77 | 82 | 61 | 76 | 77 | 70 | 100 | 114 |
| 0,1 µM | 80 | 62 | 40 | 55 | 65 | 51 | 93 | 132 |
| 10 µM | 75 | 47 | 39 | 44 | 56 | 40 | 62 | 86 |
| *13-cis retinoic acid* | | | | | | | | |
| control | 100 | 100 | 100 | 100 | 100 | 100 | 100 | 100 |
| 1 nM | 78 | 62 | 51 | 82 | 77 | 76 | 118 | 92 |
| 0,1 µM | 83 | 70 | 52 | 59 | 70 | 67 | 79 | 130 |
| 10 µM | 76 | 45 | 40 | 36 | 53 | 40 | 43 | 53 |
| *TTNPB*[a] | | | | | | | | |
| control | 100 | 100 | 100 | 100 | 100 | 100 | 100 | 100 |
| 1 nM | 80 | 100 | 51 | 67 | 85 | 63 | 92 | 96 |
| 0,1 µM | 72 | 77 | 41 | 68 | 67 | 63 | 86 | 107 |
| 10 µM | 10 | 41 | 37 | 35 | 39 | 50 | 49 | 89 |
| *TTNPB-ethylester*[b] | | | | | | | | |
| control | 100 | 100 | 100 | 100 | 100 | 100 | 100 | 100 |
| 1 nM | 77 | 88 | 50 | 63 | 82 | 80 | 103 | 80 |
| 0,1 µM | 75 | 72 | 44 | 65 | 98 | 66 | 98 | 113 |
| 10 µM | 73 | 57 | 57 | 56 | 83 | 72 | 107 | 101 |
| *TTNN*[c] | | | | | | | | |
| control | 100 | 100 | 100 | 100 | 100 | 100 | 100 | 100 |
| 1 nM | 80 | 69 | 48 | 78 | 71 | 80 | 97 | 118 |
| 0,1 µM | 84 | 54 | 43 | 72 | 62 | 69 | 92 | 151 |
| 10 µM | 84 | 59 | 52 | 41 | 26 | 66 | 57 | 119 |

[a](E)-4-[2-(5,6,7,8-tetrahydro-5,5,8,8,-tetramethyl-2-naphthalenyl)-1-propenyl] benzoic acid. [b](E)-4-[2-(5,6,7,8-tetrahydro-5,5,8,8,-tetramethyl-2-naphthalenyl)-1-propenyl] benzoic acid ethylester. [c]5′,6′,7′,8′-tetrahydro-5′,5′,8′,8′-tetramethyl-[2,2′-binaphthalene]-6-carboxylic acid.

PCR products a semi-nested PCR was used as a second step. For semi-nested PCR one slightly degenerated 5′-primer was used for amplification of all three receptor subtypes due to the high homology in this region. With 3′-primers already used in the first PCR experiment three defined PCR products for RAR-α, -β and -γ were amplified as shown in Table I. Semi-nested PCR products were separated on a 2% agarose gel as shown in Figures 2, 3 and 4. As negative control amplified RNA samples were amplified under the same conditions to confirm whether there were contaminations between the two PCR experiments.

In a third step PCR products from the semi-nested experiment were digested with specific restriction endonucleases. The RAR-α PCR product was digested with AvaI. Three defined fragments (127, 237 and 371 base pairs) separated on a 2% agarose gel are shown in Figure 5. HinfI was chosen for digestion of the RAR-β PCR product (47, 157 and 468 base pairs) as shown in Figure 6. BsmAI was used for restriction analysis of the RAR-γ PCR product. Three defined fragments (37, 97 and 364 base pairs) separated on a 2% agarose gel are shown in Figure 7.

### *Expression of RAR-α, -β* and -γ mRNA

In eight ovarian carcinoma cell lines, the epithelial ovarian cancer cell lines HOC-7, HEY, H134, HTB 77, HTB 75, OVCAR-3 and TR 170 and the ovarian teratocarcinoma cell line PA-1 the expression of RAR mRNA was studied by means of RT-PCR. For the analysis of RAR mRNA expression cells were cultivated in medium with and without 10 µM

**Figure 1** Dose response of the epithelial ovarian cancer cell line H134 **a**, and HOC-7 **b**. (1) all-*trans* retinoic acid, (2) 13-*cis* retinoic acid, (3) (E)-4-[2-(5,6,7,8-tetrahydro-5,5,8,8,-tetramethyl-2-naphthalenyl)-1-propenyl] benzoic acid (TTNPB), (4) (E)-4-[2-(5,6,7,8-tetrahydro-5,5,8,8,-tetramethyl-2-naphthalenyl)-1-propenyl] benzoic acid ethylester (TTNPB-ethylester) (5) 5′,6′,7′,8′-tetrahydro-5′,5′,8′,8′-tetramethyl-[2,2′-binaphthalene]-6-carboxylic acid (TTNN).

**Figure 2** Semi-nested PCR products of RAR-α (735 bp) separated on a 2% agarose gel, stained with ethidiumbromide. **a**, HOC-7 (1), HEY (2), H134 (3), HTB 77 (4), 100 bp DNA ladder (5), HTB 75 (6), OVCAR-3 (7), TR 170 (8), PA-1 (9). **b**, negative RNA control: HOC-7, (1), HEY (2), H134 (3), HTB 77 (4), HTB 75 (5), 100 bp DNA ladder (6), OVCAR-3 (7), TR 170 (8), PA-1 (9), $H_2O$ (10). The DNA ladder (*ordinate*) consists of 15 blunt ended fragments between 100 and 1500 base pairs in multiples of 100 bp.

**Figure 3** Semi-nested PCR products of RAR-β (672 bp) separated on a 2% agarose gel, stained with ethidiumbromide. All cells were cultivated in α-MEM containing 10 μM RA for 4 days. **a**, HOC-7 (1), HEY (2), H134 (3), HTB 77 (4), HTB 75 (5), 100 bp DNA ladder (6) OVCAR-3 (7), TR 170 (8), PA-1 (9). (**b**) Negative RNA control: HOC-7 (1), HEY (2), H134 (3), HTB 77 (4), HTB 75 (5), OVCAR-3 (6), 100 bp DNA ladder (7), TR 170 (8), PA-1 (9), $H_2O$ (10). The DNA ladder (*ordinate*) consists of 15 blunt ended fragments between 100 and 1500 base pairs in multiples of 100 bp.

**Figure 4** Semi-nested PCR products of RAR-γ (498 bp) separated on a 2% agarose gel, stained with ethidiumbromide. **a**, HOC-7 (1), HEY (2), H134 (3), HTB 77 (4), HTB 75 (5), 100 bp DNA ladder (6) OVCAR-3 (7), TR 170 (8), PA-1 (9). (**b**) Negative RNA control: HOC-7 (1), HEY (2), H134 (3), HTB 77 (4), HTB 75 (5), 100 bp DNA ladder (6), OVCAR-3 (7), TR 170 (8), PA-1 (9), $H_2O$ (10). The DNA ladder (*ordinate*) consists of 15 blunt ended fragments between 100 and 1500 base pairs in multiples of 100 bp.

all-*trans* RA. All cell lines investigated expressed RAR-α and -γ mRNA under both conditions. Whereas RAR-α and -γ mRNA showed a high baseline expression (Figure 2 and 4), RAR-β mRNA expression could be demonstrated at a significant level only under stimulation with RA; except in HOC-7 and OVCAR-3 cells where RAR-β mRNA was not detectable at all (Figure 3; data without RA stimulation of RAR-β mRNA expression are not shown).

### Discussion

Differentiation therapy may become an additional or even alternative therapeutical approach for the management of cancer beyond the actual conventional cytotoxic treatment. Retinoids belong to a group of substances appropriate for therapeutical use. Since short time retinoids represent the treatment of choice in acute promyelocytic leukaemia. In

**Figure 5** Restriction endonuclease digestion of the RAR-α semi-nested PCR products separated on a 2% agarose gel, stained with ethidiumbromide (fragments: 127, 237 and 371 bp). HOC-7 (1), HEY (2), H134 (3), 100 bp DNA ladder (4), HTB 77 (5), HTB 75 (6), OVCAR-3 (7), TR 170 (8), 100 bp DNA ladder (9), PA-1 (10). The DNA ladder (*ordinate*) consists of 15 blunt ended fragments between 100 and 1500 base pairs in multiples of 100 bp.

**Figure 6** Restriction endonuclease digestion of the RAR-β semi-nested PCR products separated on a 2% agarose gel, stained with ethidiumbromide (fragments: 47, 157 and 468 bp). HEY (1), H134 (2), HTB 77 (3), 100 bp DNA ladder (4), HTB 75 (5), TR 170 (6), PA-1 (7). The DNA ladder (*ordinate*) consists of 15 blunt ended fragments between 100 and 1500 base pairs in multiples of 100 bp.

addition, these agents have been successfully used in the therapy of squamous cell carcinomas and in the prevention of second primary carcinomas of the aerobronchodigestive tract (Hong *et al.*, 1990; Lippman *et al.*, 1992).

Although ovarian cancer is a tumour entity responding moderately well or even well to cytotoxic chemotherapy an overall 5-year survival of approximately 30–40% is still unsatisfactory. For this reason our group intended to evaluate alternative therapeutic strategies for ovarian carcinoma, among them induction of differentiation.

The aim of our study was to investigate the effects of RA and synthetic RA-analogs on the growth of human ovarian cancer cell lines. The cell lines used in this study have been characterised previously. They differ clearly in their growth behaviour and morphology. While HOC-7, HEY, H134 and PA-1 are rapidly growing cells, the cell lines HTB 77, HTB 75, OVCAR-3 and TR 170 represent a slower growing cell type (Hamilton *et al.*, 1983; Buick *et al.*, 1985; Hill *et al.*, 1987; Broxterman *et al.*, 1987). All epithelial ovarian cancer cell lines were inhibited in their growth by RA and synthetic RA-analogs in a dose dependent manner. The cell lines HTB 77, HTB 75, OVCAR-3 and TR 170 were highly responsive to the retinoids tested, but also the rapidly growing cell lines HEY and especially H134 were inhibited in their growth by retinoids. The ovarian adenocarcinoma cell line HOC-7 had already been used earlier by us as a model for growth inhibition and differentiation induction by polar-planar compounds like Dimethylsulfoxide and N,N′-dimethylformamide as well as by transforming growth factor-β1 (TGF-β1) and by all-*trans* RA. But, all-*trans* RA caused only a weak growth inhibition and induction of differentiation associated antigens in this cell line (Grunt *et al.*, 1992*a*, 1992*b*; Somay *et al.*, 1992). The various retinoids tested in our experiments also yielded only weak growth inhibition in this cell line. The fact that both the slowly growing cell lines and the two rapidly growing cells HEY and H134 were intensively inhibited in their growth by retinoids led us to conclude that the response of the epithelial ovarian cancer cell lines to these agents was not directly dependent on their proliferation capacity. In contrast, the ovarian teratocarcinoma cell line PA-1 exhibited a divergent behaviour when treated with these substances. No growth inhibition, but even a growth promoting effect at nanomolar concentrations of each retinoid tested was observed.

Due to the characterisation of the nuclear RARs a better insight into the mode of action of retinoids on the target cells was gained. RAR-α seems to be distributed ubiquitously in cells and tissues, while the expression of RAR-β and -γ mRNA is tissue-specific (de Luca, 1991). Abnormal expression of RAR-β mRNA has been reported for some hepatoma cells and in human oral and epidermal squamous cell carcinoma cell lines (Hu *et al.*, 1991). RAR-γ mRNA has been shown to be abundantly expressed in the skin (Krust *et al.*, 1989). Therefore we wanted to know whether any expression of RAR mRNA could be detected in the ovarian cancer cell lines and whether there was a difference in the presence or absence of RAR subtype mRNA among them. For these investigations the method of RT-PCR was chosen as a highly sensitive tool for detecting specific gene transcripts. This method utilises the cellulary expressed mRNA as template

**Figure 7** Restriction endonuclease digestion of the RAR-γ semi-nested PCR products separated on a 2% agarose gel, stained with ethidiumbromide (fragments: 37, 97 and 364 bp). HOC-7 (1), HEY (2), 100 bp DNA ladder (3), H134 (4), HTB 77 (5), HTB 77 (6), OVCAR-3 (7), 100 bp ladder (8), TR 170 (9), PA-1 (10). The DNA ladder (*ordinate*) consists of 15 blunt ended fragments between 100 and 1500 base pairs in multiples of 100 bp.

for single-stranded cDNA synthesis that becomes PCR-amplified subsequently. PCR was performed under stringent conditions to distinguish between all three receptor subtypes and PCR products were additionally confirmed by restriction endonuclease digestion. All cell lines used in this study expressed RAR-α and -γ mRNA, but two of the eight cell lines failed to express RAR-β transcripts. As reported previously, RA and synthetic retinoids bind each receptor subtype with different affinities and cause specific transcriptional activation of target genes (Aström *et al.*, 1990; Graupner *et al.*, 1991). The lack of RAR-β mRNA in HOC-7 and OVCAR-3 cells would therefore lead us to expect a similar response of both cell lines to retinoids, but a divergent behaviour to the retinoids tested was observed among the two cell lines. On the other side, the cell line PA-1, which was non-responsive to the retinoids tested expressed mRNAs for all three receptor subtypes. Overall, we could not observe a conclusive association between the presence of RAR subtype transcripts and the response to retinoids in these cell lines. The most striking finding, that there was a lack of RAR-β transcripts in two out of eight cell lines, has to be investigated in the future on the molecular level more in depth. First, by the method of RT-PCR only a qualitative but not a quantitative determination of RAR transcripts could be performed which will be quantified by Northern Analysis and second, rearrangements or deletions in the RAR-β gene or other mutational events may be suspected to be the underlaying mechanism for the observed lack of RAR-β mRNA expression and will be the target of further investigations (Pratt *et al.*, 1990; Hu *et al.*, 1991).

We conclude that RA and synthetic retinoids are potent substances to induce growth inhibition in ovarian cancer cells. In continuation to these studies the determination of differentiation associated parameters in retinoid treated ovarian cancer cells to distinguish between growth inhibition and differentiation induction and the use of retinoids in combination with other substances in order to increase their differentiation capacity are planned.

The authors wish to thank Mrs Anna Stöger and Dr Gottfried Himmler (Institut fuer Angewandte Mikrobiologie, Universitaet fuer Bodenkultur Wien) for primer synthesis.

This work was supported by grants from the Jubilaeumsfonds der Oesterreichischen Nationalbank, the Medizinisch-Wissenschaftlicher Fonds des Buergermeister der Bundeshauptstadt Wien and by the Anton-Dreher Gedaechtnisschenkung fuer Medizinische Forschung.

### References

ASTRÖM, A., PETTERSON, U., KRUST, A., CHAMBON, P. & VOORHEES, J.J. (1990). Retinoic acid and synthetic analogs differentially activate retinoic acid receptor dependent transcription. *Biochem. Biophys. Res. Comm.*, **173**, 339-345.

BENBROOK, D., LERNHARDT, E. & PFAHL, M. (1988). A new retinoic acid receptor identified from a hepatocellular carcinoma. *Nature*, **333**, 669-672.

BRAND, N., PETKOVICH, M., KRUST, A., CHAMBON, P., DE THE, H., MARCHIO, A., TILLOIS, P. & DEJEAN, A. (1988). Identification of a second human retinoic acid receptor. *Nature*, **332**, 850-853.

BROXTERMAN, H.J., SPRENKELS-SCHOTTE, C., ENGELEN, P.H., LEYVA, A. & PINEDO, H.M. (1987). Analysis of human ascites effect on clonogenic growth of human tumor cell lines and NRK-49F cells in soft agar. *Int. J. Cell Clon.*, **5**, 158-169.

BUICK, R.N., PULLANO, R. & TRENT, J.M. (1985). Comparative properties of five human ovarian adenocarcinoma cell lines. *Canc. Res.*, **45**, 3668-3676.

CHOMCZYNSKI, P. & SACCHI, N. (1987). Single-step method of RNA isolation by acid guanidinium-thiocyanate-phenol-choloroform extraction. *Anal. Biochem.*, **162**, 156-159.

DE LUCA, L.M. (1991). Retinoids and their receptors in differentiation, embryogenesis, and neoplasia. *FASEB J.*, **5**, 2924-2933.

DE THE, H., VIVANCO-RUIZ, M., TIOLLAIS, P., STUNNENBERG, H. & DEJEAN, A. (1990). Identification of a retinoic acid response element in the retinoic acid receptor β gene. *Nature*, **343**, 177-180.

DELECLUSE, C., CAVEY, M.T., MARTIN, B., BERNARD, B.A., REICHERT, U., MAIGNAN, J., DARMON, M. & SHROOT, B. (1991). Selective high affinity retinoic acid receptor α or β-γ ligands. *Mol. Pharmacol.*, **40**, 556-562.

EICHELE, G. (1989). Retinoids and vertebrate limb pattern formation. *Trends. Genet.*, **5**, 246-251.

GIGUERE, V., ONG, E.S., SEGUI, P. & EVANS, R.M. (1987). Identification of a receptor for the morphogen retinoic acid. *Nature*, **330**, 624-629.

GLASS, C.K., LIPKIN, S.M., DEVARY, O.V. & ROSENFELD, M.G. (1989). Positive and negative regulation of gene transcription by a retinoic acid-thyroid hormone receptor heterodimer. *Cell*, **59**, 697-708.

GRAUPNER, G., MALLE, G., MAIGNAN, J., LANG, G., PRUNIERAS, M. & PFAHL, M. (1991). 6′-substituted naphthalene-2-carboxylic acid analogs, a new class of retinoic acid receptor subtype-specific ligands. *Biochem. Biophys. Res. Comm.*, **179,** 1554–1561.

GRUNT, TH. W., SOMAY, C., ELLINGER, A., PAVELKA, M., DITTRICH, E. & DITTRICH, CH. (1992*a*). The differential effects of N,N′-dimethylformamide and transforming growth factor-β1 on a human ovarian cancer cell line (HOC-7). *J. Cell Physiol.*, **151,** 13–22.

GRUNT, TH. W., SOMAY, C., OELLER, H., DITTRICH, E. & DITTRICH, CH. (1992*b*). Comparative analysis of the effects of dimethylsulfoxide and retinoic acid on the antigenic pattern of human ovarian adenocarcinoma cells. *J. Cell Sci.*, **103,** 501–509.

HAMILTON, TH. C., YOUNG, R.C., MCKOY, W.M., GROTZINGER, K.R., GREEN, J.A., CHU, E.W., WHANG-PENG, J., ROGAN, A.M., GREEN, W.R. & OZOLS, R.F. (1983). Characterization of a human ovarian carcinoma cell line (NIH:OVCAR-3) with androgen and estrogen receptors. *Canc. Res.*, **43,** 5379–5389.

HEYMAN, R.A., MANGELSDORF, D.J., DYCK, J.A., STEIN, R.B., EICHELE, G., EVANS, R.M. & THALLER, C.H. (1992). 9-cis retinoic acid is a high affinity ligand for the retinoid X receptor. *Cell*, **68,** 397–406.

HILL, B.T., WHELAN, R.D.H., GIBBY, E.M., SHEER, D., HOSKING, L.K., SHELLARD, S.A. & RUPNIAK, H. TH. (1987). Establishment and characterisation of three human ovarian carcinoma cell lines and initial evaluation of their potential in experimental chemotherapy studies. *Int. J. Cancer*, **39,** 219–225.

HONG, W.K., LIPPMAN, S.M., ITRI, L.M., KARP, D.D., LEE, J.S., BYERS, R.M., SCHANTZ, S.P., KRAMER, A.M., LOTAN, R., PETERS, L.J., DIMERY, I.W., BROWN, B.W. & GOEPFERT, H. (1990). Prevention of second primary tumors with isotretinoin in squamous-cell carcinoma of the head and neck. *N. Eng. J. Med.*, **323,** 795–801.

HU, L., CROWE, D.L., RHEINWALD, J.G., CHAMBON, P. & GUDAS, L.J. (1991). Abnormal expression of retinoic acid receptors and keratin 19 by human oral and epidermal squamous carcinoma cell lines. *Canc. Res.*, **51,** 3972–3981.

KRUST, A., KASTNER, PH., PETKOVICH, M., ZELENT, A. & CHAMBON, P. (1989). A third human retinoic acid receptor, hRAR-γ. *Proc. Natl Acad. Sci. USA*, **86,** 5310–5314.

LIPPMAN, S.M. & MEYSKENS, F.L. Jr. (1989). Results of the use of vitamin A and retinoids in cutaneous malignancies. *Pharmacol. Ther.*, **40,** 107–122.

LIPPMAN, S.M., PARKINSON, D.R., ITRI, L.M., WEBER, R.S., SCHANTZ, S.P., OTA, D.M., SCHUSTERMAN, M.A., KRAKOFF, I.H., GUTTERMAN, J.U. & HONG, W.K. (1992). 13-cis retinoic acid and interferon α-2a: Effective combination therapy for advanced squamous cell carcinoma of the skin. *J. Natl Cancer Inst.*, **84,** 235–241.

MANGELSDORF, D.J., ONG, E.S., DYCK, J.A. & EVANS, R.M. (1990). Nuclear receptor that identifies a novel retinoic acid response pathway. *Nature*, **345,** 224–229.

MENG-ER, H., YU-CHEN, Y., SHU-RONG, C., JIN-REN, C., JIA-XIANG, L., LIN, Z., LONG-JUN, G. & ZHEN-YI, W. (1988). Use of all-trans retinoic acid in the treatment of acute promyelocytic leukemia. *Blood*, **72,** 567–572.

MOSMANN, T. (1983). Rapid colorimetric assay for cellular growth and survival: Application to proliferation and cytotoxicity assays. *J. Immunol. Meth.*, **65,** 55–63.

MULLIS, K.B. & FALOONA, F.A. (1987). Specific synthesis of DNA *in vitro* via a polymerase catalyzed reaction. *Method. Enzymol.*, **155,** 335–350.

PETKOVICH, M., BRAND, N.J., KRUST, A. & CHAMBON, P. (1987). A human retinoic acid receptor which belongs to the family of nuclear receptors. *Nature*, **330,** 444–450.

PRATT, M.A., KRALOVA, J. & MCBURNEY, M.W. (1990). A dominant negative mutation of the alpha retinoic acid receptor gene in a retinoic acid non-responsive embryonal carcinoma. *Mol. Cell Biol.*, **10,** 6445–6453.

SAIKI, R.K., GELFAND, D.H., STOFFEL, S., SCHARF, S.J., HIGUCHI, R., HORN, G.T., MULLIS, K.B. & ERLICH, H.A. (1988). Primer-directed enzymatic amplification of DNA with a thermostable DNA polymerase. *Science*, **239,** 487–491.

SOMAY, C., GRUNT, TH. W., MANNHALTER, CH. & DITTRICH, CH. (1992). Relationship of myc protein expression to the phenotype and to the growth potential of HOC-7 ovarian cancer cells. *Br. J. Cancer*, **66,** 93–98.

SPORN, M.B., ROBERTS, A.B. & GOODMAN, D.S. (Eds) (1984). *The Retinoids*, Vol. 1–2, Academic Press, New York.

UMESONO, K., GIGUERE, V., GLASS, C.K., ROSENFELD, M.G. & EVANS, R.M. (1988). Retinoic acid and thyroid hormone induce gene experession through a common responsive element. *Nature*, **336,** 262–265.

YU, V.C., DELSERT, C., ANDERSEN, B., HOLLOWAY, J.M., DEVARY, O.V., NÄÄR, A.M., KIM, S.Y., BOUTIN, J.M., GLASS, C.K. & ROSENFELD, M.G. (1991). RXRβ: A coregulator that enhances binding of retinoic acid, thyroid hormone, and vitamin D receptors to their cognate response elements. *Cell*, **67,** 1251–1266.